# Awareness and Perception of Telemedicine Among the General Population in the Central, Northern, and Western Regions of Saudi Arabia

**DOI:** 10.7759/cureus.64895

**Published:** 2024-07-19

**Authors:** Mohamed M Abd El Mawgod, Atheer A Alshutayli, Sultan M Alanazi, Wahaj N Alqahtani, Nada A Alqahtani, Ammar M Alamri, Nouf Z Alshammari

**Affiliations:** 1 Family and Community Medicine, Northern Border University, Arar, SAU; 2 Public Health and Community Medicine, AlAzhar University, Assiut, EGY; 3 College of Medicine, Qassim University, Buraydah, SAU; 4 College of Medicine, Northern Border University, Arar, SAU; 5 College of Medicine, King AbdulAziz University, Jeddah, SAU; 6 College of Medicine, King Khalid University, Abha, SAU

**Keywords:** health care delivery, telemedicine, saudi arabia, perception, awareness

## Abstract

Background: Telemedicine is a paradigm shift that utilizes technology for remote healthcare delivery, improving the quality of care.

Objectives: This study aims to assess the general population's awareness and perception regarding telemedicine in the central, northern, and western regions of Saudi Arabia.

Methods: A web-based cross-sectional study was conducted in the central, northern, and western regions of Saudi Arabia from January 2024 to May 2024. A structured questionnaire was used to collect data, including sociodemographic information and questions to assess participants' awareness and perception of telemedicine. Data were analyzed using IBM SPSS Statistics for Windows, Version 27 (Released 2020; IBM Corp., Armonk, New York, United States), with significance at p < 0.05.

Results: Out of 414 adult participants in the study, 264 (63.8%) were female, and 205 (49.5%) were below the age of 25. Approximately a quarter of the participants reported being very or moderately familiar with telemedicine. Furthermore, most participants (80.5%, 243) expressed their willingness to try telemedicine. The most common barriers to telemedicine practice were concerns about diagnostic reliability, resistance from physicians, and patient resistance. The study found no significant associations between participants' sociodemographic variables, their familiarity with telemedicine, and their readiness to adopt it.

Conclusion: While there is a low level of awareness and knowledge regarding telemedicine among general populations in Saudi Arabia, there is generally a positive perception and willingness to adopt telemedicine for improved healthcare delivery. Addressing barriers to adopting such technology is crucial to ensure the country's successful implementation and widespread acceptance of telemedicine.

## Introduction

Technology has rapidly developed and remarkably grown in various sectors contributing to a knowledge-driven economy. Hence, it has emphasized the significance of increased investment in mobile phone infrastructure and establishing e-government initiatives. In the medical industry, electronic technology has expanded its use in the healthcare sector through the emergence of telemedicine [1].

Telemedicine, defined as the remote use of online e-resources in the delivery of healthcare services, has transformed traditional patient-doctor interactions by enabling diagnosis and treatment to occur over long distances, eliminating the need for in-person appointments [2]. As a versatile tool, telemedicine has demonstrated success across multiple health-related domains, including screening, education, research, and direct healthcare provision [3]. Its applications span from managing infectious diseases like COVID-19 to addressing chronic conditions such as diabetes and hypertension, facilitating rehabilitation efforts, and involving caretakers in caring for persons with disabilities [4]. Furthermore, mental health services have witnessed an expanding shift toward teleservices, incorporating various methods such as text-based communication and videoconferencing, to better cater to individuals needing support [5].

Harvard Medical School delineates a spectrum of advantages and drawbacks associated with telemedicine. Among the administrative benefits are the efficient management of measurements, notes, and alerts, alongside the facilitation of convenient and prompt medical appointments. Financially, telemedicine offers potential savings on healthcare services and transportation costs, while enhancing medical benefits through expedited access to healthcare providers and online medical results. Hence, telemedicine has been viewed as a cost-effective solution to tackle the obstacles in healthcare delivery, especially for individuals living in remote regions or experiencing limited availability of medical services. Telemedicine has been viewed as a promising solution to address healthcare delivery challenges, particularly for individuals residing in remote areas or with limited access to medical services [6,7]. However, notable challenges include concerns over security and privacy, restricted health insurance coverage for specific telehealth services, and the potential risk of services being exclusively confined to online platforms [8].

The global tendency toward electronic monitoring of health has expanded. Developed countries showed expanded growth in electronic technology use in health-related fields [9]. Around 65% of US phone users have downloaded at least one healthcare application [10]. On the contrary, the concept of telemedicine remains novel and unclear in developing countries [3]. The adoption of telemedicine in Saudi Arabia is relatively new and received a notable boost during COVID-19. In response, The Sehhaty platform and the 937 Call Center, initiated by the National Ministry of Health, have been introduced to enhance telemedicine services throughout the kingdom. These initiatives operate around the clock, offering convenient access to healthcare and serving as valuable additions to existing e-health programs [11].

Low utilization hinders wide adoption of this technology in the community [12,13]. A growing body of literature has examined factors that influence the adoption of telemedicine services in both developed and developing countries. These determinants encompass factors such as patients' acceptance and trust, attitudes of healthcare providers, regulatory frameworks, and technological infrastructure [6,12,14]. Good knowledge and a positive perception of telemedicine is crucial for the high-quality implementation of this technology [15]. Despite the dissemination of telemedicine in Saudi Arabia, a notable concentration on telemedicine within the context of COVID-19 may not entirely reflect the general population's awareness and attitude toward telemedicine under normal circumstances [16]. Therefore, it is essential to promote knowledge and cultivate a positive attitude toward telemedicine among the Saudi Arabian population, even outside the context of the COVID-19 pandemic. Additionally, addressing any concerns or misconceptions about telemedicine can help alleviate apprehensions and build trust in the technology [17].

Despite the recognized significance of telemedicine in diagnostic testing, decision-making, staff training, and data management, there remain several gaps in research within this field. It is crucial to comprehend the perceptions and satisfaction levels of the general public to optimize the implementation of telemedicine and effectively address the growing healthcare demands. Evaluating the public's acceptance and awareness of telemedicine would serve as a valuable tool in promoting its adoption and enhancing the quality and safety of telemedicine practices. Hence, this study aims to assess the awareness and perception of telemedicine among the general population residing in the central, northern, and western regions of Saudi Arabia.

## Materials and methods

Study setting and design

A multi-center cross-sectional study was conducted in the central, northern, and western regions of Saudi Arabia from January 2024 to May 2024.

Study population

Adults at least 18 years old, living in the central, northern, or western regions of Saudi Arabia, and accepted to participate were eligible to be enrolled in the study.

Sampling method

Using a convenience sampling method, a survey was conducted for the enrolment of participants from the central, western, and northern regions by online questionnaire method utilizing different social media platforms (WhatsApp, Twitter, Facebook).

Sample size

The minimum sample size was estimated using the following formula [18].

$\text{Sample size (N)} = \frac{(Z_{1-\alpha/2})^2 P (1-P)}{d^2} = \frac{(1.96)^2 \times 0.55 \times (0.45)}{(0.05)^2} = 380.1 \approx 381$

Where $Z_{1-\alpha/2}$ is the standard normal variate at 5% type 1 error (1.96), P is the expected proportion of awareness in Saudi Arabia (55%) from a previous study [16], and d is the absolute error (0.05). We increased it to 414 to compensate for the non-response rate.

Study tool

A structured questionnaire prepared by reviewing the relevant literature was used [16]. The first part includes data about sex, age, marital status, education level, and occupation. The second part of the questionnaire involved inquiry to assess awareness regarding telemedicine. The participants were questioned about their familiarity with the term "telemedicine" and their awareness of communicative medical services offered in their community. They were also asked about their previous encounters with telemedicine services, the specific timing of their first use of telemedicine, the reasons prompting them to utilize telemedicine (if applicable), their level of knowledge regarding telemedicine tools, and their preferred choices when it comes to telemedicine tools. In the third section, participants' perception regarding telemedicine was assessed through a series of questions. These questions aimed to gauge participants' readiness to try telemedicine, their belief in telemedicine as an effective tool for providing patient care, their satisfaction with virtual consultations, their perception of telemedicine as a cost-effective option, their opinion on whether telemedicine saves time, their view on the diagnostic concordance of telemedicine compared to direct mutual consultations, and their belief that telemedicine can decrease the need to outpatient care. Additionally, participants were asked about their thoughts on whether the effectiveness of telemedicine depends on the specialty and if telemedicine can be used to monitor chronic patients from home. Furthermore, participants were asked to identify barriers to the practice of telemedicine from a list of options, including physician resistance, patient resistance, diagnostic reliability, cultural aspects, and technological problems.

Statistical analysis

Data entry and subsequent data analysis were conducted using IBM SPSS Statistics for Windows, Version 27 (Released 2020; IBM Corp., Armonk, New York, United States). To ensure data quality, incomplete and inconsistent participant data were excluded. Qualitative data were presented as numbers and percentages. Pearson's chi-square test (χ^2^) for independence was used to measure differences between groups. In cases where the assumptions of the chi-square test were not met, the Monte Carlo test was employed as an alternative. Descriptive measures, such as means and standard deviations, were used to present numerical variables. All statistical tests were two-tailed, and statistical significance was assessed using a p-value threshold of 0.05.

## Results

Table 1 presents the socio-demographic profile of the participants. Among the studied population, 63.8% were females, 49.5% were below 25 years old, 66.7% were single, 57.7% were university graduates, and 42% were students. In terms of Saudi regions, the participants were mostly from the central province (42.8%), with the western province and northern province accounting for 28.0% and 29.2%, respectively.

**Table 1:** Socio-demographic profile of the studied participants

Sociodemographic variables		n (414)	%
Age (years)	Mean ± SD 29.7 ± 11.4		
Age groups	<25	205	49.5
	25-34	102	24.6
	35-44	41	9.9
	45-54	50	12.1
	≥55	16	3.8
Sex	Male	150	36.2
	Female	264	63.8
Saudi region	Central province	177	42.8
	Western province	116	28.0
	Northern province	121	29.2
Marital status	Married	125	30.2
	Single	276	66.7
	Divorced	7	1.7
	Widowed	6	1.4
Education level	Not educated	2	0.5
	Primary school	4	1.0
	Middle school	7	1.7
	High school	121	29.2
	University graduate	239	57.7
	Postgraduate	41	9.9
Occupation	Student	174	42.0
	Not employed	69	16.7
	Governmental employed	84	20.3
	Private	48	11.6
	Free jobs	15	3.6
	Retired	24	5.8

Table 2 shows the participants' awareness of telemedicine. Regarding familiarity with telemedicine term, approximately a quarter of the participants reported being very familiar (24.6%) or moderately familiar (24.6%) with telemedicine. In terms of awareness of telemedicine services in the community, 56.6% of participants indicated that they were aware of such services. Regarding utilization of telemedicine services, 46.4% of participants reported having used telemedicine services. A significant proportion of those who had used it (83.6%) reported using it after the COVID-19 pandemic. The most common reasons for their usage were medical consultations (51.4%), follow-up appointments (7.1%), prescription of medication (6.4%), and multiple uses (35.1%). Regarding knowledge of telemedicine tools, 33.6% were aware of video or phone calls, 12.9% were familiar with chat-based telemedicine, and 53.6% reported knowing all of the mentioned tools. Regarding the preferred telemedicine tools, 40.0% preferred video or phone calls, while 21.4% favored chat-based telemedicine, and 38.6% expressed a preference for all of the mentioned tools.

**Table 2:** Awareness of telemedicine among the studied participants *: some questions were not answered by all participants as is not applicable

Awareness items		n^*^	%
Familiarity with telemedicine term	Very familiar	102	24.6
	Moderate familiar	102	24.6
	Slightly familiar	98	23.7
	Not familiar at all	112	27.1
Knowledge of the existence of any communicative medicine services offered in the local community	Yes	180	56.6
	No	122	40.4
Ever used telemedicine services	Yes	140	46.4
	No	162	53.6
The first use of telemedicine	After COVID-19	117	83.6
	Before COVID-19	23	16.4
If used, please choose the reasons for using telemedicine	Medical consultations	72	51.4
	Follow up	10	7.1
	Prescription of medication	9	6.4
	More than one use	49	35.1
Knowing telemedicine tools	Video or phone calls	47	33.6
	Chat	18	12.9
	All of above	75	53.6
Preferred telemedicine tools	Video or phone calls	56	40.0
	Chat	30	21.4
	All of above	54	38.6

Table 3 illustrates participants' perceptions of telemedicine. In terms of readiness to try telemedicine, the majority of participants (80.5%) expressed their willingness to try it for diagnosis and follow-up. Regarding the effectiveness of telemedicine in remote patient care, 35.1% agreed and 41.7% strongly agreed with this statement. Participants' satisfaction with virtual consultations was generally positive, with 42.4% agreeing and 33.1% strongly agreeing that patients are satisfied with virtual consultations. Regarding the cost-effectiveness of telemedicine, almost half of the participants (49.3%) strongly agreed that telemedicine is cost-effective, while 37.1% agreed. In terms of time-saving potential, 57.3% strongly agreed that telemedicine saves time, with an additional 32.8% agreeing. Compared to in-person direct consultations, 23.5% of participants agreed and 20.5% strongly agreed that telemedicine shows good diagnostic concordance. Participants also recognized the potential of telemedicine to reduce unnecessary outpatient visits, with 33.4% agreeing and 57% strongly agreeing with this statement. When asked about the effectiveness of telemedicine depending on the specialty, 37.4% disagreed and 40.7% strongly disagreed that telemedicine's effectiveness is specialty-dependent. Regarding the use of telemedicine for monitoring chronic patients from home, 38.1% of participants agreed and 40.4% strongly agreed with this statement. Regarding barriers to the practice of telemedicine, the reported barriers were diagnostic reliability (40.7%) and physician resistance (18.9%).

**Table 3:** Perception toward telemedicine among the studied participants Note: data presented as number and %

Perception items		n = 302 (%)
Readiness to use telemedicine for diagnostic and follow-up purposes	Yes	243 (80.5)
	I don't know	47 (15.5)
	No	12 (4.0)
Telemedicine is an effective tool for providing patient care	Strongly agree	126 (41.7)
	Agree	106 (35.1)
	Neutral	65 (21.5)
	Disagree	4 (1.3)
	Strongly disagree	1 (0.3)
Satisfaction with online consultations	Strongly agree	100 (33.1)
	Agree	128 (42.4)
	Neutral	64 (21.2)
	Disagree	7 (2.3)
	Strongly disagree	3 (1.0)
Cost-effectiveness of telemedicine	Strongly agree	149 (49.3)
	Agree	112 (37.1)
	Neutral	33 (10.9)
	Disagree	4 (1.3)
	Strongly disagree	4 (1.3)
Saving time is an advantage of telemedicine	Strongly agree	173 (57.3)
	Agree	99 (32.8)
	Neutral	22 (7.3)
	Disagree	4 (1.3)
	Strongly disagree	4 (1.3)
Telemedicine demonstrates a favorable level of diagnostic concordance when compared to in-person consultations	Strongly agree	62 (20.5)
	Agree	71 (23.5)
	Neutral	89 (29.5)
	Disagree	60 (19.9)
	Strongly disagree	20 (6.6)
Telemedicine has the potential to decrease unnecessary outpatient visits	Strongly agree	172 (57.0)
	Agree	101 (33.4)
	Neutral	24 (7.9)
	Disagree	3 (1.0)
	Strongly disagree	2 (0.7)
The effectiveness of telemedicine depends on the specialty	Strongly agree	3 (1.0)
	Agree	4 (1.3)
	Neutral	59 (19.5)
	Disagree	113 (37.4)
	Strongly disagree	123 (40.7)
Telemedicine enables remote monitoring of patients with chronic conditions	Strongly agree	122 (40.4)
	Agree	115 (38.1)
	Neutral	42 (13.9)
	Disagree	18 (6.0)
	Strongly disagree	5 (1.7)
Barriers to telemedicine implementation	Physician resistance	57 (18.9)
	Patient resistance	21 (7.0)
	Diagnostic reliability	123 (40.7)
	Cultural aspects	28 (9.3)
	Technological problems	39 (9.4)
	Others	4 (1.3)
	More than one	30 (9.9)

Table 4 presents the association between participants' familiarity with the concept of telemedicine and their sociodemographic profile. There were insignificant associations between familiarity with telemedicine and participants' sociodemographic profile variables, including sex, age, Saudi region, marital status, education level, and occupation (p>0.05).

**Table 4:** Association between familiarity with telemedicine term and participants’ sociodemographic profile χ^2^: chi-square test; MC: Monte Carlo test

Sociodemographic profile		Familiarity with the telemedicine term				p-value
		Not familiar at all n 112 (%)	Slightly familiar n 98 (%)	Moderately familiar n 102 (%)	Very familiar n 102 (%)	
Sex	Male	38 (33.9)	37(37.8)	36 (35.3)	39 (38.2)	^χ2^p=0.904
	Female	74 (66.1)	61 (62.2)	66 (64.7)	63 (61.8)	
Age (years)	<25	61 (54.5)	41 (41.8)	48 (47.1)	55 (53.9)	^MC^p=0.303
	25-34	26 (23.2)	24 (24.5)	23 (22.5)	29 (28.4)	
	35-44	11 (9.8)	9 (9.2)	14 (13.7)	7 (6.9)	
	45-54	11 (9.8)	18 (18.4)	12 (11.8)	9 (8.8)	
	≥55	3 (2.7)	6 (6.1)	5 (4.9)	2 (2.0)	
Saudi region	Central province	52 (46.4)	39 (39.8)	37 (36.3)	49 (48.0) 26 (25.5) 27 (26.5)	^χ2^p=0.355
	Western province	27 (24.1)	34 (34.7)	29 (28.4)		
	Northern province	33 (29.5)	25 (25.5)	36 (35.3)		
Marital status	Married	28 (25.0)	36 (36.7)	35 (34.3)	26 (25.5) 76 (74.5) 0 (0.0) 0 (0.0)	^MC^p=0.147
	Single	79 (70.5)	58 (59.2)	63 (61.8)		
	Divorced	3 (2.7)	3 (3.1)	1 (1.0)		
	Widowed	2 (1.8)	1 (1.0)	3 (2.9)		
Education level	Not educated	1 (0.9)	0 (0.0)	0 (0.0)	1 (1.0)	^MC^p=0.406
	Primary school	1 (0.9)	2 (2.0)	1 (1.0)	0 (0.0)	
	Middle school	3 (2.7)	1 (1.0)	1 (1.0)	2 (2.0)	
	High school	32 (28.6)	30 (30.6)	23 (22.5)	36 (35.3)	
	University graduate	70 (62.5)	54 (55.1)	65 (63.7)	50 (49.0)	
	Postgraduate	5 (4.5)	11 (11.1)	12 (11.8)	13 (12.7)	
Occupation	Student	44 (39.3)	41 (41.8)	43 (42.2)	46 (45.1)	^χ2^p=0.766
	Not employed	26 (23.2)	14 (14.3)	13 (12.7)	16 (15.7)	
	Governmental employed	19 (17.0)	20 (20.4)	22 (21.6)	23 (22.5)	
	Private	13 (11.6)	11 (11.3)	13 (12.7)	11 (10.8)	
	Free Jobs	6 (5.4)	4 (4.1)	4 (3.9)	1 (1.0)	
	Retired	4 (3.6)	8 (8.2)	7 (6.9)	5 (4.9)	

Table 5 presents the association between participants' readiness to try telemedicine and their sociodemographic profile. Readiness to try telemedicine didn’t show significant associations with participants' sociodemographic variables, including sex, age, Saudi region, education level, marital status, and occupation (p>0.05).

**Table 5:** Association between readiness to try telemedicine and participants’ sociodemographic χ^2^: chi-square test; MC: Monte Carlo test

Sociodemographic profile		Readiness to try telemedicine			p-value
		No n 12(%)	I don’t know n 47(%)	Yes n 243(%)	
Sex	Male	4 (33.3)	15 (31.9)	93 (38.3)	^χ2^p=0.685
	Female	8 (66.7)	32 (68.1)	150 (61.7)	
Age (years)	<25	7 (58.3)	28 (59.6)	109 (44.9)	^MC^p=0.208
	25-34	2 (16.7)	10 (21.3)	64 (26.3)	
	35-44	3 (25.0)	2 (4.3)	25 (10.3)	
	45-54	0 (0.0)	4 (8.5)	35 (14.4)	
	≥55	0 (0.0)	3 (6.4)	10 (4.1)	
Saudi region	Central province	5 (41.7)	22 (46.8)	98 (40.3)	^MC^p=0.732
	Western province	2 (16.7)	13 (27.7)	74 (30.5)	
	Northern province	5 (41.7)	12 (25.5)	71 (29.2)	
Marital status	Married	4 (33.3)	17 (36.2)	76 (31.3)	^MC^p=0.531
	Single	8 (66.7)	27 (57.4)	162 (66.7)	
	Divorced	0 (0.0)	1 (2.1)	3 (1.2)	
	Widowed	0 (0.0)	2 (4.3)	2 (0.8)	
Education level	Not educated	0 (0.0)	0 (0.0)	1 (0.4)	^MC^p=0.992
	Primary school	0 (0.0)	0 (0.0)	3 (1.2)	
	Middle school	0 (0.0)	1 (2.1)	3 (1.2)	
	High school	4 (33.3)	12 (25.5)	73 (30.0)	
	University graduate	7 (58.3)	28 (59.6)	134 (55.1)	
	Postgraduate	1 (8.3)	6 (12.8)	29 (11.9)	
Occupation	Student	7 (58.3)	21 (44.7)	102 (42.0)	^MC^p=0.719
	Not employed	3 (25.0)	8 (17.0)	32 (13.2)	
	Governmental employed	1 (8.3)	9 (19.1)	55 (22.6)	
	Private	0 (0.0)	5 (10.6)	30 (12.3)	
	Free jobs	1 (8.3)	1 (2.1)	7 (2.9)	
	Retired	0 (0.0)	3 (6.4)	17 (7.0)	

## Discussion

The primary focus of adopting telemedicine in Saudi Arabia is to improve the quality and accessibility of healthcare services, with a specific focus on patients residing in rural and remote regions. This technology-driven approach seeks to bridge the gap between patients and healthcare providers, enabling remote consultations, diagnosis, and treatment options, thereby enhancing the overall healthcare experience [16]. Awareness and perception regarding telemedicine are important aspects to be studied, particularly after the COVID-19 pandemic. Telemedicine has become a crucial tool for providing healthcare services, serving as a valuable resource to inform future implementation strategies and optimize rollout approaches [19].

The general population’s awareness of telemedicine was explored in this study from different angles. The study revealed that about a quarter of participants were familiar with telemedicine, while almost half had utilized telemedicine services, particularly after the COVID-19 pandemic. Medical consultations were the primary reason for usage. Participants showed greater awareness of video or phone calls, followed by chat-based telemedicine. Video or phone calls were the preferred modality, followed by chat-based telemedicine, with a significant proportion expressing a preference for all tools. Regarding perception, participants displayed a positive perception of telemedicine, with the majority expressing willingness to try it for diagnosis and follow-up. They also believed that telemedicine is an effective method for remote patient care, cost-effective, time-saving, and shows good diagnostic concordance. Participants recognized the potential of telemedicine to reduce unnecessary outpatient visits and to monitor chronic patients from home. Overall, among adult Saudi Arabians residing in the central, northern, and western regions, there was low awareness of telemedicine. However, most participants displayed a positive attitude toward telemedicine and expressed readiness to utilize it for various healthcare needs. Several studies conducted in Saudi Arabia support these findings. Talmesany et al. [16], Albarrak et al. [7], Alnajrani et al. [20], and Mubaraki et al. [21] have all recorded a limited understanding of telemedicine but a generally positive perception regarding its adoption. Talmesany et al. [16], who conducted their study among the Al-Baha population found that familiarity with telemedicine services was low, with only 54.9% utilizing telemedicine services. Despite this, participants expressed a positive perception of telemedicine services. Additionally, Albarrak et al. [7], reported that a significant number of medical professionals in Riyadh, Saudi Arabia, exhibited low awareness of telemedicine. However, most participants showed a positive attitude toward telemedicine and expressed willingness to utilize it. On the other hand, in another study involving Saudi citizens, Alnajrani et al. [20], found a positive attitude among the general public toward telepharmacy services. It is important to acknowledge that the support and motivation from users play a crucial role in transforming technology into a practical solution. This support enables the system to expand its reach and enhances the value that health professionals bring to the field of e-health [22].These results highlight the need for increased public knowledge regarding the significance and effectiveness of this emerging technology.

Sociodemographic disparities in awareness and perception of telemedicine were further examined in this study. Insignificant associations were found between participants' familiarity with telemedicine or their readiness to try it and different socio-demographic factors. Similarly, Talmesany et al. [16] and Darrat et al. [23] found that sociodemographic factors did not correlate with awareness and perception of telemedicine. Hence, the impact of demographic factors may not significantly influence the adoption of telemedicine. This finding implies that certain populations, who are considered vulnerable, may not engage in telehealth services, despite their ongoing healthcare needs in an evolving healthcare delivery environment. Furthermore, community health education to different population subgroups is important to raise awareness and improve the utilization of telemedicine.

The study identified various barriers to the adoption of telemedicine, which encompassed concerns regarding the reliability of diagnoses, resistance from both physicians and patients, cultural factors, and technological issues. Additionally, limited availability, privacy, and security concerns, as well as apprehensions about the quality of care, were identified as significant obstacles hindering the utilization of telemedicine services [16]. Additionally, Albarrak et al. [7] reported that there were barriers to telemedicine adoption including lack of privacy and training, high cost, and technological challenges. Mubaraki et al. [21]. also concluded that although physicians acknowledged the effectiveness of telemedicine in enhancing the quality of care, several concerns were identified as barriers to telemedicine adoption [7]. Furthermore, El Kheir et al. [24] showed that physicians who worked at King Fahad Hospital reported that legal issues with telemedicine regulations hinder its application, suggesting that small percentages of physicians adhere to the guidelines. Overcoming these barriers is essential to ensure the fruitful integration and acceptance of telemedicine in the future. A key strategy to address these challenges is the implementation of high-quality education and training programs. Hence, healthcare providers and the general population can enhance their understanding and proficiency in utilizing telemedicine effectively. This would not only improve the delivery of healthcare services but also enable individuals to make informed decisions regarding their healthcare needs. Furthermore, legislators should take action to pass laws that address and eliminate obstacles related to technology accessibility, technical literacy, and connectivity [25].

Study limitations

Although the study has exhibited valued information regarding the awareness and perception of the general population in different regions of Saudi Arabia, there are limitations. The study's limitations include potential sampling bias from the use of a web-based survey. Additionally, self-reporting bias may influence participants' responses.

## Conclusions

Among adult Saudi Arabians residing in central, northern, and western regions, there was a low awareness level regarding telemedicine. However, the perception of telemedicine among the participants was positive. Concerns about diagnostic reliability, resistance from physicians and patients, cultural aspects, and technological problems were reported as barriers to the widespread practice of telemedicine. Interestingly, the study did not find significant associations between participants' familiarity with telemedicine or their willingness to try it and various socio-demographic factors. In light of these findings, it is crucial to address the identified barriers and promote telemedicine as a viable option for healthcare delivery in Saudi Arabia. Strategies should focus on increasing awareness, providing education and training, addressing concerns related to diagnostic reliability, and improving technological infrastructure. By leveraging these favorable attitudes and addressing the identified challenges, Saudi Arabia can pave the way for successful implementation and acceptance of telemedicine, thus transforming healthcare delivery and improving patient outcomes.
